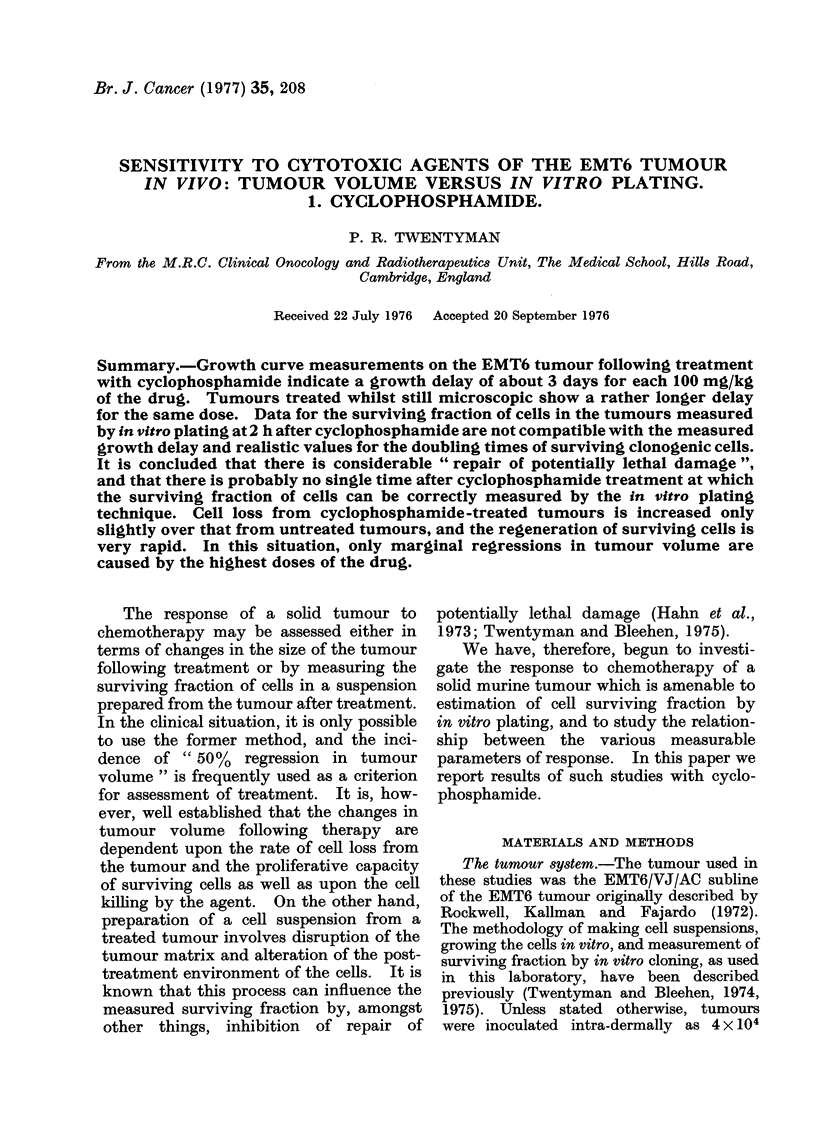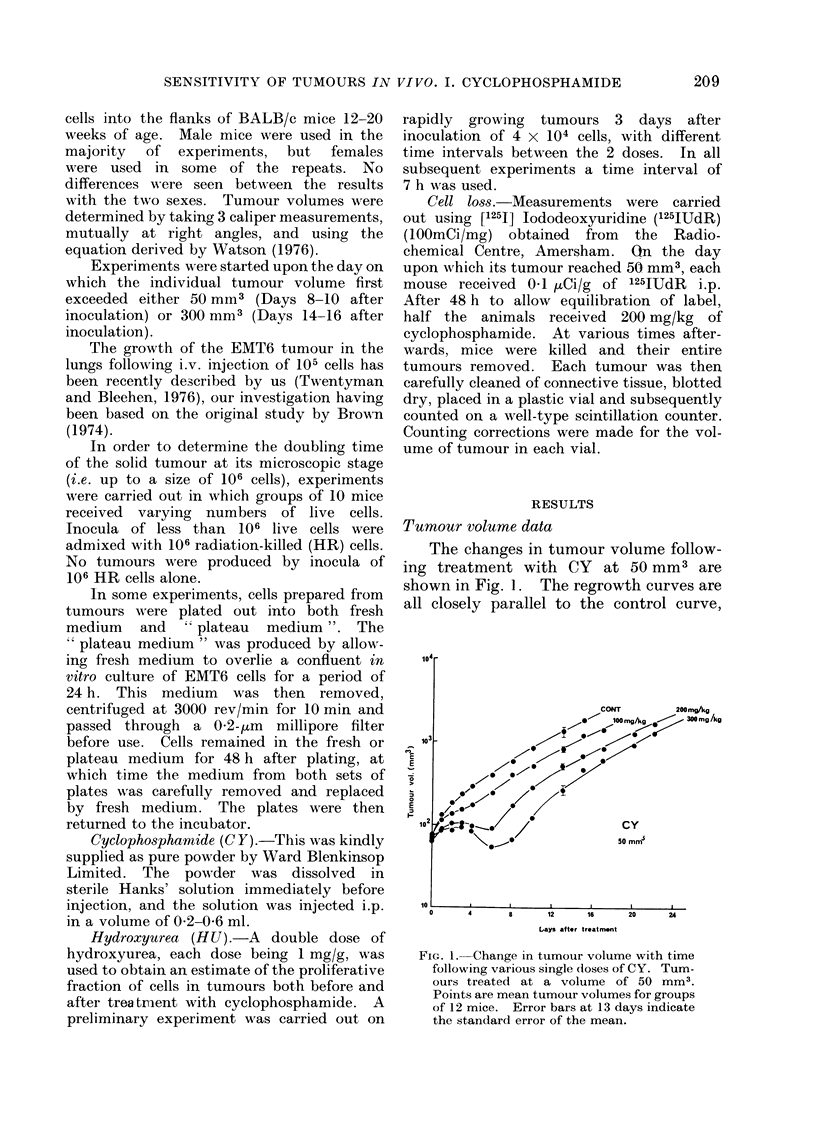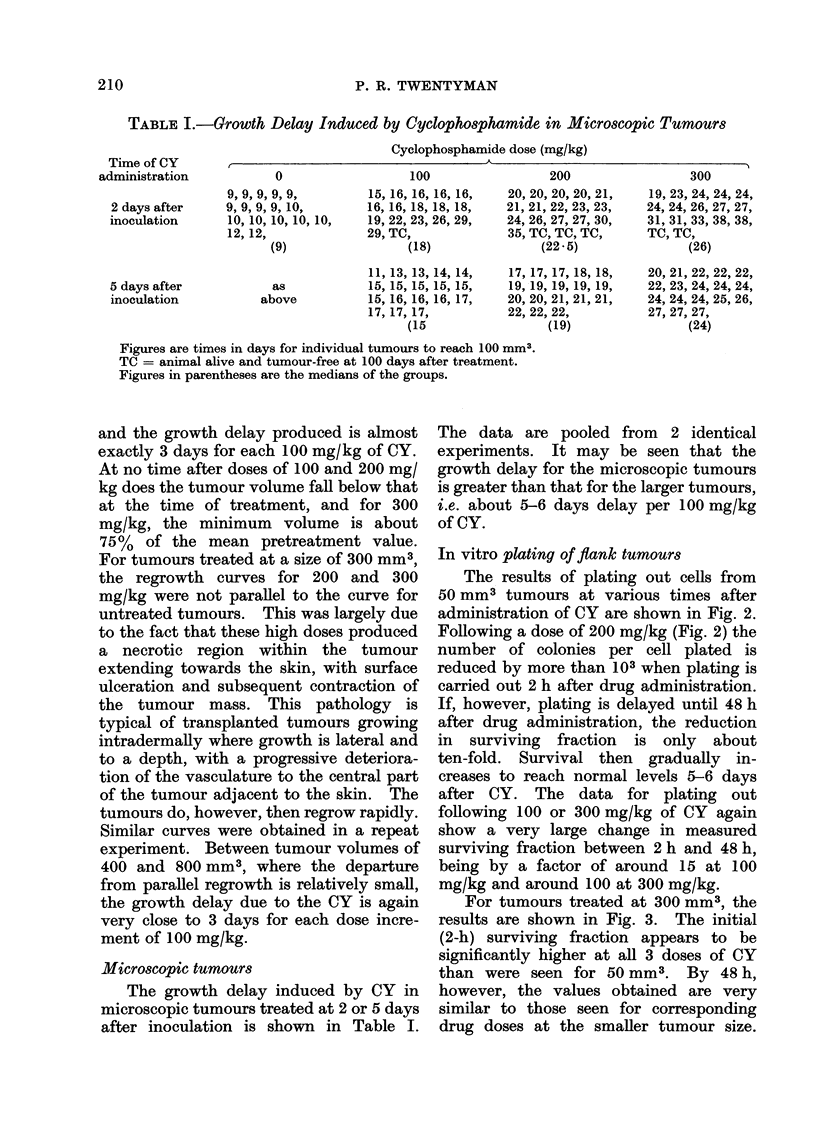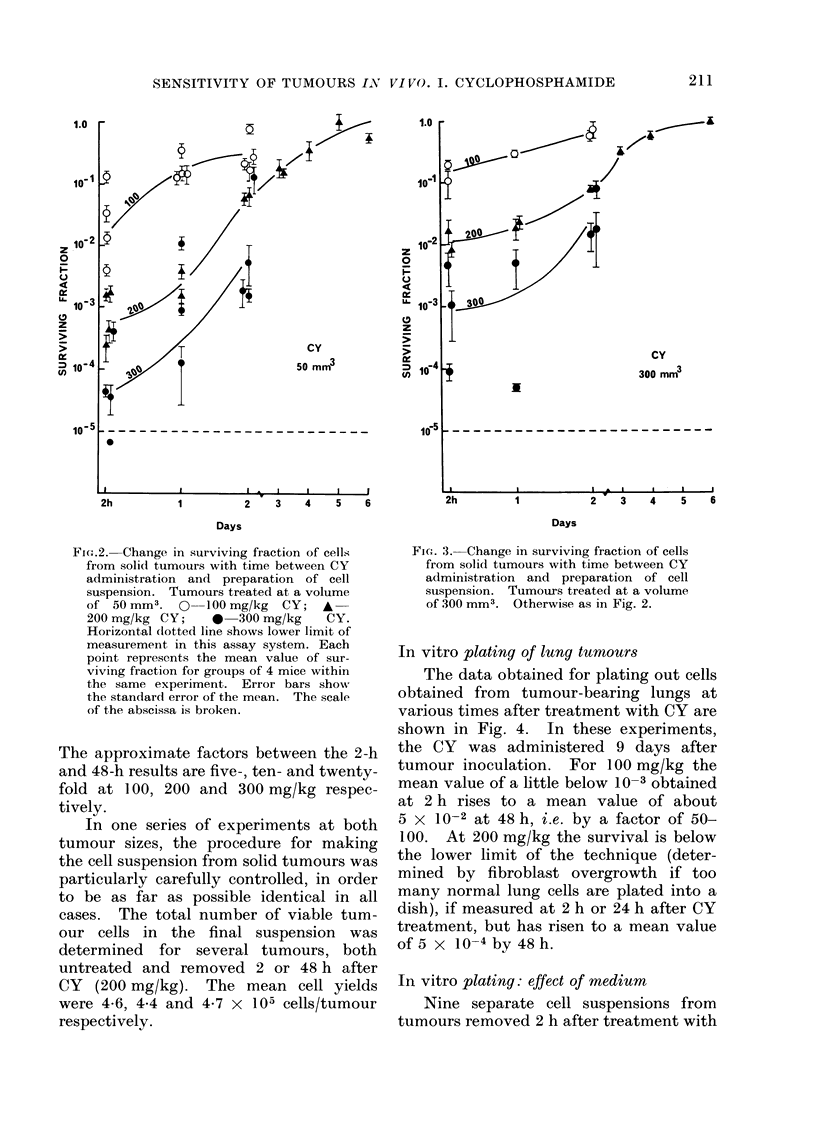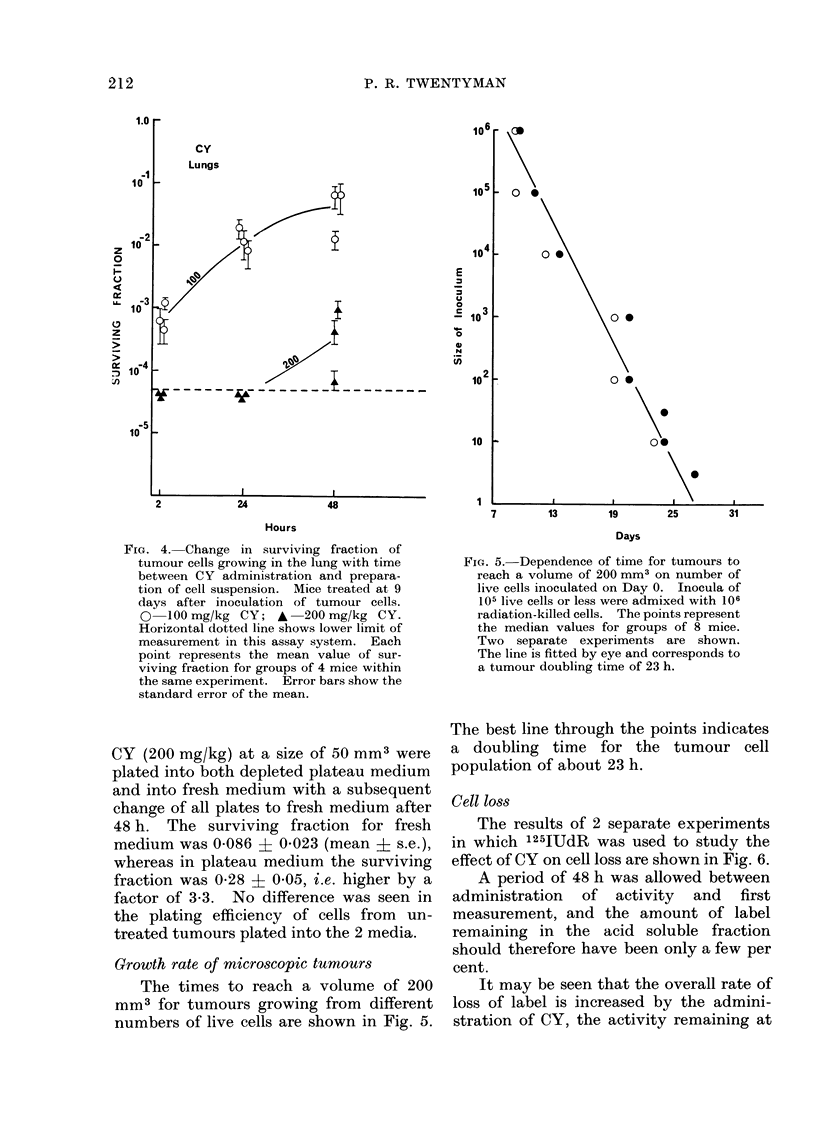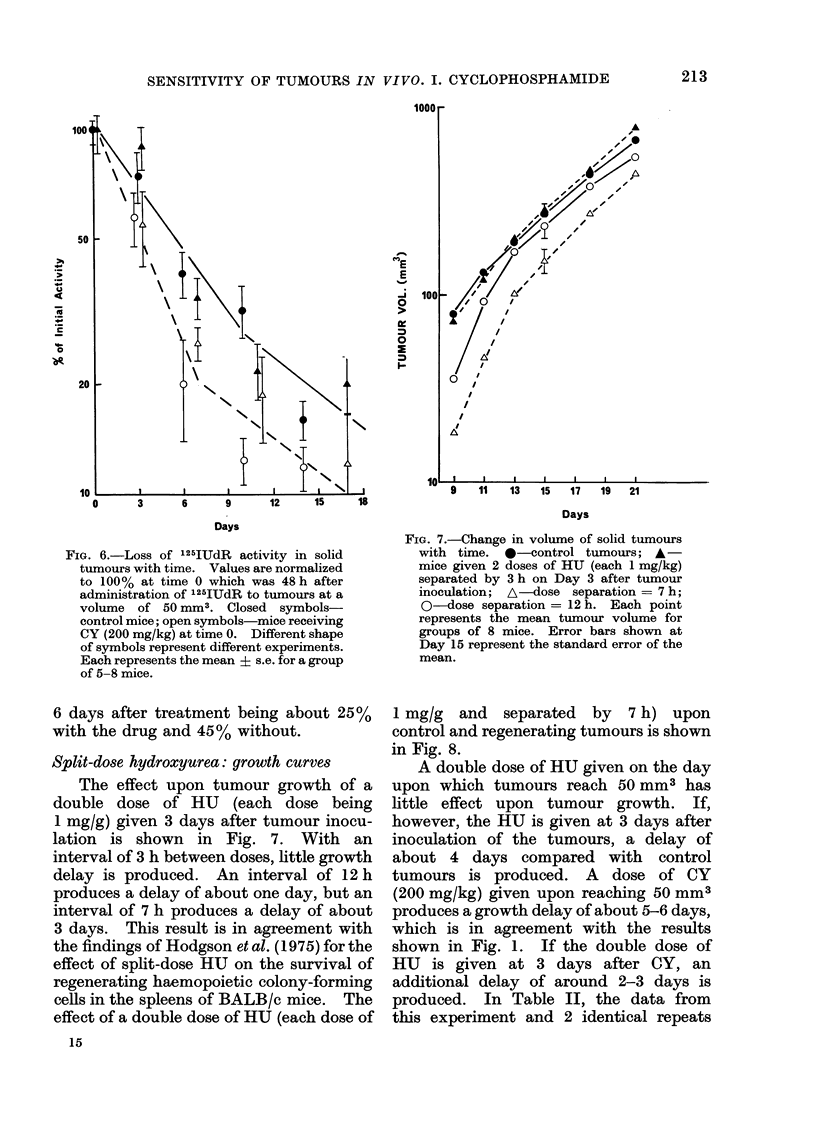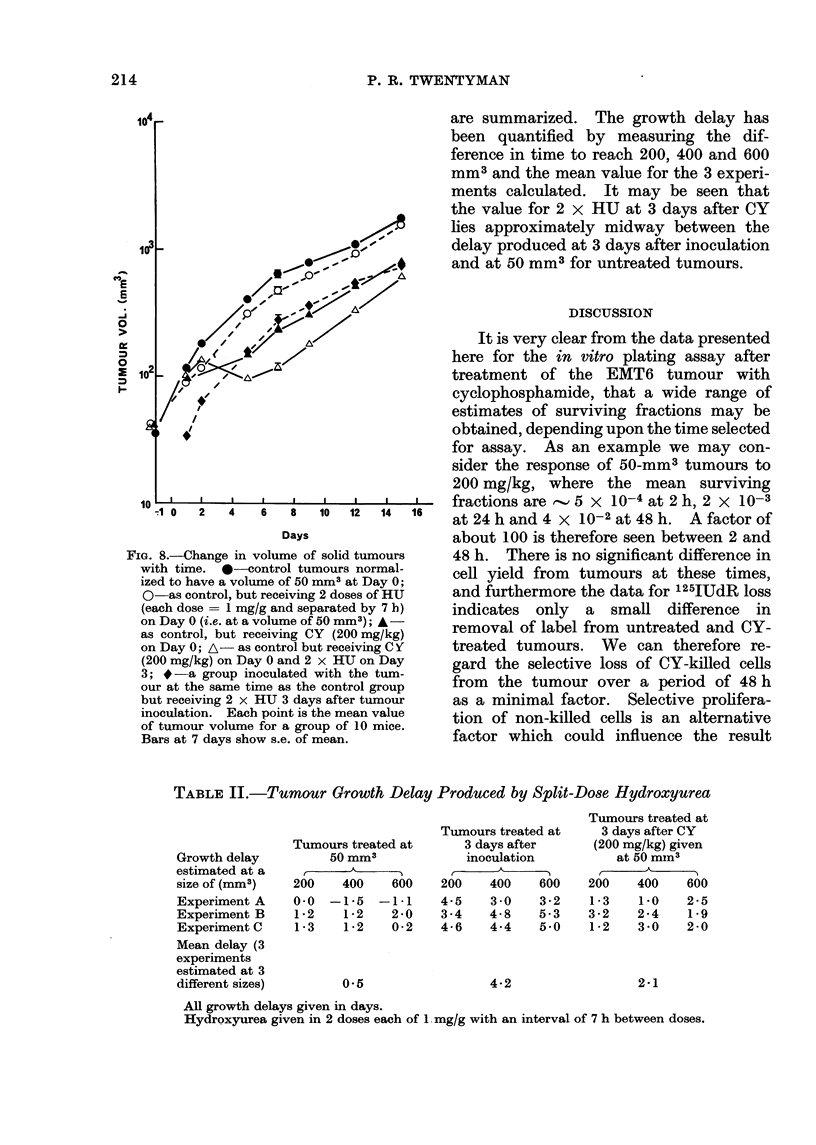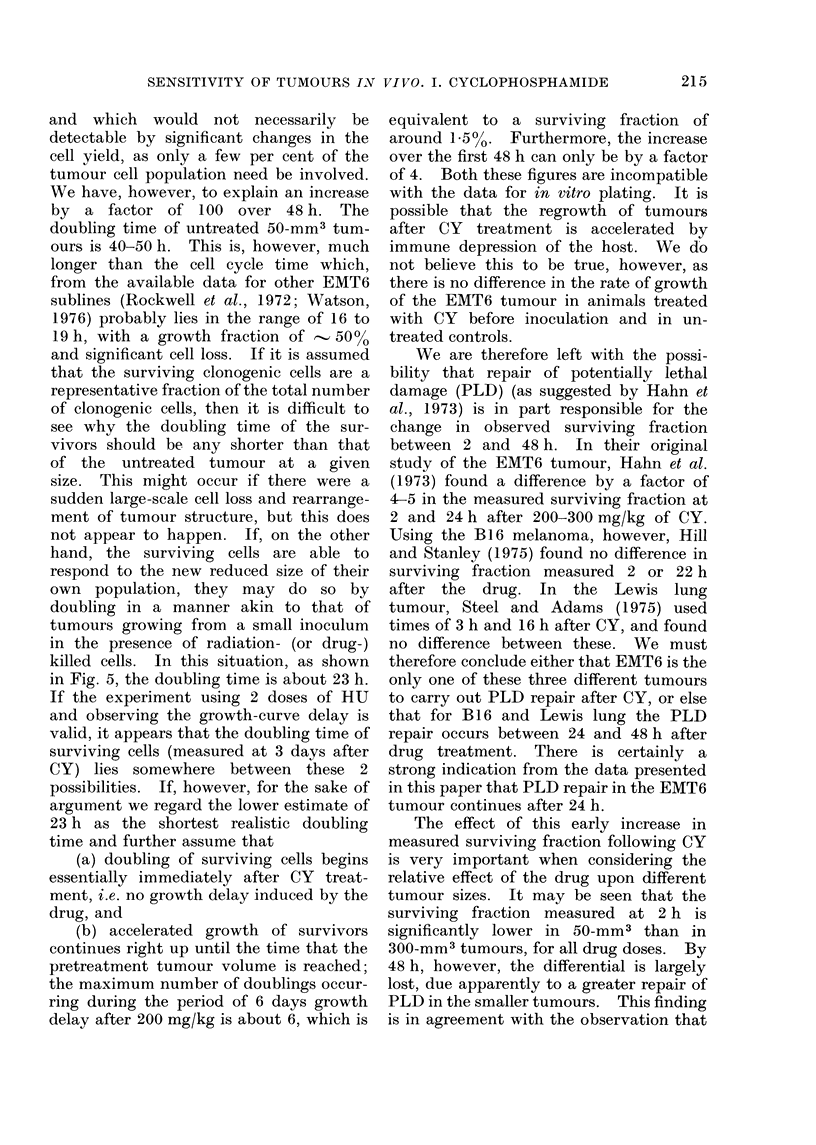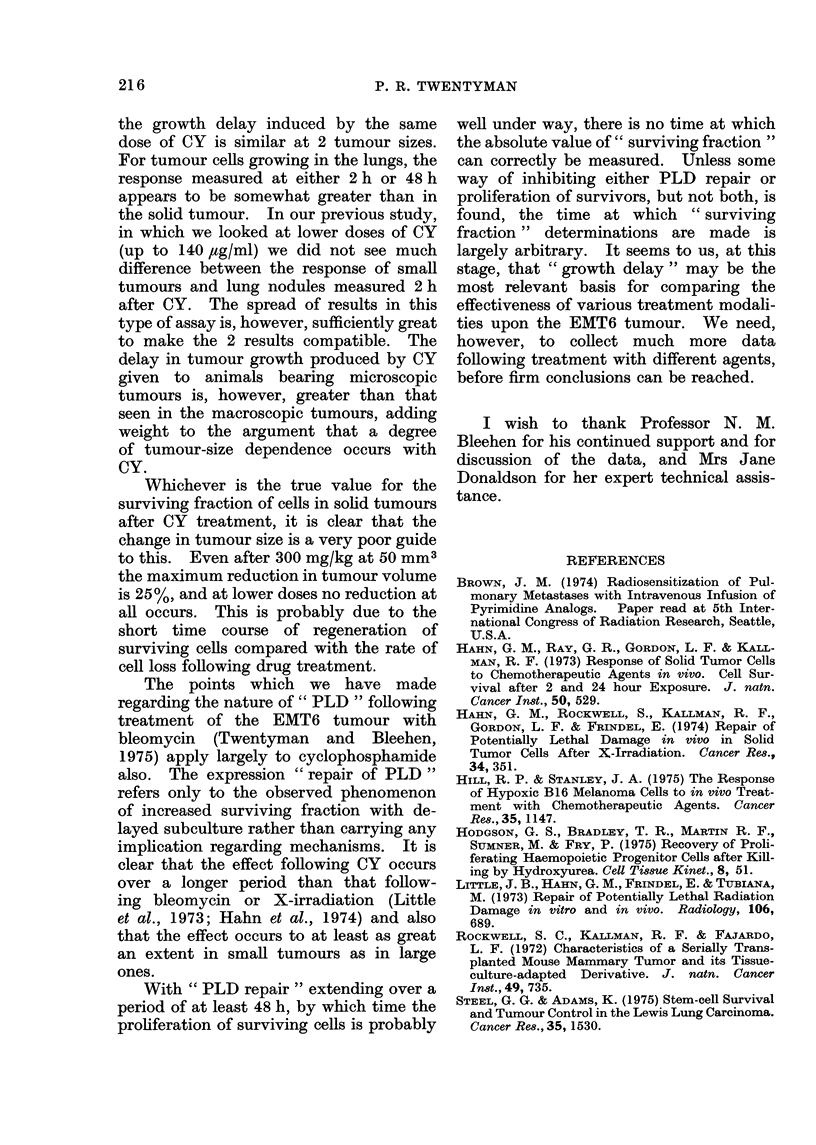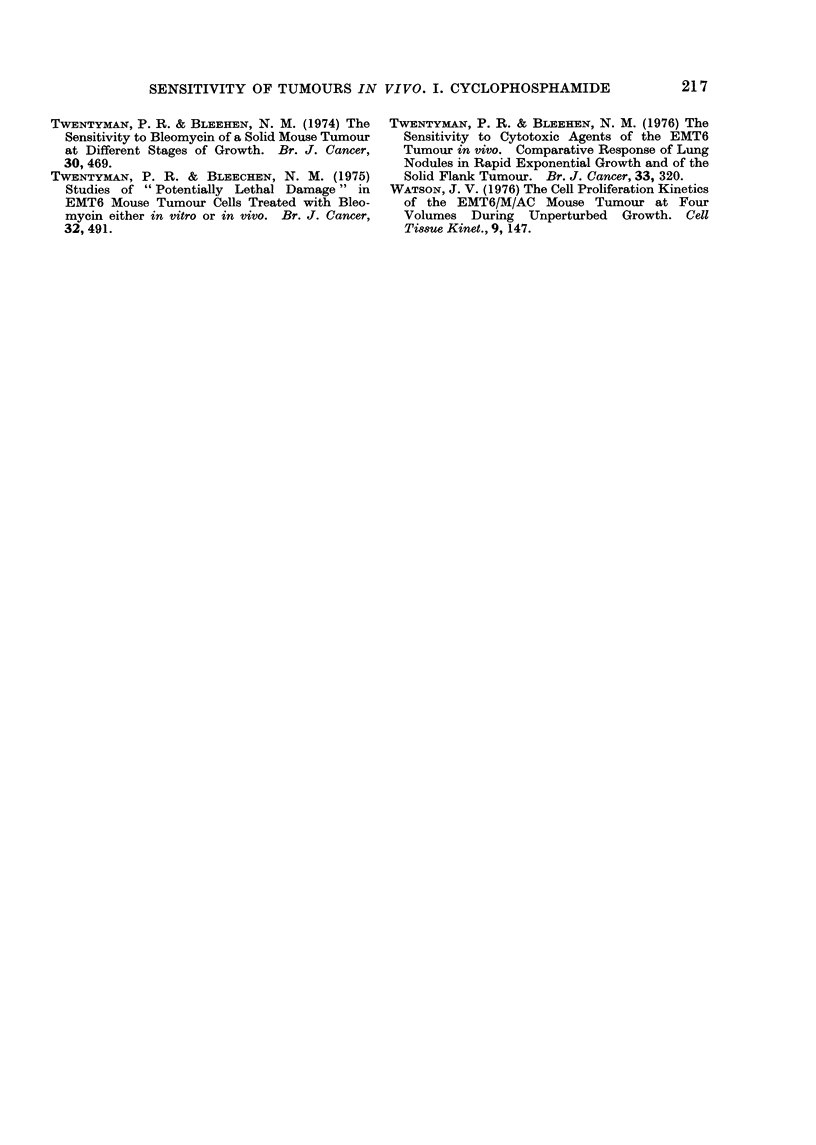# Sensitivity to cytotoxic agents of the EMT6 tumour in vivo: tumour volume versus in vitro plating. 1. Cyclophosphamide.

**DOI:** 10.1038/bjc.1977.28

**Published:** 1977-02

**Authors:** P. R. Twentyman

## Abstract

Growth curve measurements on the EMT6 tumour following treatment with cyclophosphamide indicate a growth delay of about 3 days for each 100 mg/kg of the drug. Tumours treated whilst still microscopic show a rather longer delay for the same dose. Data for the surviving fraction of cells in the tumours measured by in vitro plating at 2 h after cyclophosphamide are not compatible with the measured growth delay and realistic values for the doubling times of surviving clonogenic cells; It is concluded that there is considerable "repair of potentially lethal damage", and that there is probably no single time after cyclophosphamide treatment at which the surviving fraction of cells can be correctly measured by the in vitro plating technique. Cell loss from cyclophosphamide-treated tumours is increased only slightly over that from untreated tumours, and the regeneration of surviving cells is very rapid. In this situation, only marginal regressions in tumour volume are caused by the highest doses of the drug.


*Br. J. Cancer* (1977) **35**, 208

### SENSITIVITY TO CYTOTOXIC AGENTS OF THE EMT6 TUMOUR *IN VIVO*: TUMOUR VOLUME VERSUS *IN VITRO* PLATING. 1. CYCLOPHOSPHAMIDE.

P. R. TWENTYMAN

*From the M.R.C. Clinical Onocology and Radiotherapeutics Unit, The Medical School, Hills Road, Cambridge, England*

Received 22 July 1976 Accepted 20 September 1976

**Summary.—Growth curve measurements on the EMT6 tumour following treatment with cyclophosphamide indicate a growth delay of about 3 days for each 100 mg/kg of the drug. Tumours treated whilst still microscopic show a rather longer delay for the same dose. Data for the surviving fraction of cells in the tumours measured by *in vitro* plating at 2 h after cyclophosphamide are not compatible with the measured growth delay and realistic values for the doubling times of surviving clonogenic cells. It is concluded that there is considerable " repair of potentially lethal damage ", and that there is probably no single time after cyclophosphamide treatment at which the surviving fraction of cells can be correctly measured by the *in vitro* plating technique. Cell loss from cyclophosphamide-treated tumours is increased only slightly over that from untreated tumours, and the regeneration of surviving cells is very rapid. In this situation, only marginal regressions in tumour volume are caused by the highest doses of the drug.**

The response of a solid tumour to chemotherapy may be assessed either in terms of changes in the size of the tumour following treatment or by measuring the surviving fraction of cells in a suspension prepared from the tumour after treatment. In the clinical situation, it is only possible to use the former method, and the incidence of " 50% regression in tumour volume " is frequently used as a criterion for assessment of treatment. It is, however, well established that the changes in tumour volume following therapy are dependent upon the rate of cell loss from the tumour and the proliferative capacity of surviving cells as well as upon the cell killing by the agent. On the other hand, preparation of a cell suspension from a treated tumour involves disruption of the tumour matrix and alteration of the post-treatment environment of the cells. It is known that this process can influence the measured surviving fraction by, amongst other things, inhibition of repair of potentially lethal damage (Hahn *et al.*, 1973; Twentyman and Bleehen, 1975).

We have, therefore, begun to investigate the response to chemotherapy of a solid murine tumour which is amenable to estimation of cell surviving fraction by *in vitro* plating, and to study the relationship between the various measurable parameters of response. In this paper we report results of such studies with cyclophosphamide.

### MATERIALS AND METHODS

*The tumour system.*—The tumour used in these studies was the EMT6/VJ/AC subline of the EMT6 tumour originally described by Rockwell, Kallman and Fajardo (1972). The methodology of making cell suspensions, growing the cells *in vitro*, and measurement of surviving fraction by *in vitro* cloning, as used in this laboratory, have been described previously (Twentyman and Bleehen, 1974, 1975). Unless stated otherwise, tumours were inoculated intra-dermally as $4 \times 10^4$

cells into the flanks of BALB/c mice 12–20 weeks of age. Male mice were used in the majority of experiments, but females were used in some of the repeats. No differences were seen between the results with the two sexes. Tumour volumes were determined by taking 3 caliper measurements, mutually at right angles, and using the equation derived by Watson (1976).

Experiments were started upon the day on which the individual tumour volume first exceeded either 50 mm$^3$ (Days 8–10 after inoculation) or 300 mm$^3$ (Days 14–16 after inoculation).

The growth of the EMT6 tumour in the lungs following i.v. injection of $10^5$ cells has been recently described by us (Twentyman and Bleehen, 1976), our investigation having been based on the original study by Brown (1974).

In order to determine the doubling time of the solid tumour at its microscopic stage (*i.e.* up to a size of $10^6$ cells), experiments were carried out in which groups of 10 mice received varying numbers of live cells. Inocula of less than $10^6$ live cells were admixed with $10^6$ radiation-killed (HR) cells. No tumours were produced by inocula of $10^6$ HR cells alone.

In some experiments, cells prepared from tumours were plated out into both fresh medium and " plateau medium ". The " plateau medium " was produced by allowing fresh medium to overlie a confluent *in vitro* culture of EMT6 cells for a period of 24 h. This medium was then removed, centrifuged at 3000 rev/min for 10 min and passed through a 0·2-$\mu$m millipore filter before use. Cells remained in the fresh or plateau medium for 48 h after plating, at which time the medium from both sets of plates was carefully removed and replaced by fresh medium. The plates were then returned to the incubator.

*Cyclophosphamide* (*CY*).—This was kindly supplied as pure powder by Ward Blenkinsop Limited. The powder was dissolved in sterile Hanks' solution immediately before injection, and the solution was injected i.p. in a volume of 0·2–0·6 ml.

*Hydroxyurea* (*HU*).—A double dose of hydroxyurea, each dose being 1 mg/g, was used to obtain an estimate of the proliferative fraction of cells in tumours both before and after treatment with cyclophosphamide. A preliminary experiment was carried out on rapidly growing tumours 3 days after inoculation of 4 × $10^4$ cells, with different time intervals between the 2 doses. In all subsequent experiments a time interval of 7 h was used.

*Cell loss*.—Measurements were carried out using [$^{125}$I] Iododeoxyuridine ($^{125}$IUdR) (100mCi/mg) obtained from the Radiochemical Centre, Amersham. On the day upon which its tumour reached 50 mm$^3$, each mouse received 0·1 $\mu$Ci/g of $^{125}$IUdR i.p. After 48 h to allow equilibration of label, half the animals received 200 mg/kg of cyclophosphamide. At various times afterwards, mice were killed and their entire tumours removed. Each tumour was then carefully cleaned of connective tissue, blotted dry, placed in a plastic vial and subsequently counted on a well-type scintillation counter. Counting corrections were made for the volume of tumour in each vial.

### RESULTS

### *Tumour volume data*

The changes in tumour volume following treatment with CY at 50 mm$^3$ are shown in Fig. 1. The regrowth curves are all closely parallel to the control curve,

FIG. 1.—Change in tumour volume with time following various single doses of CY. Tumours treated at a volume of 50 mm$^3$. Points are mean tumour volumes for groups of 12 mice. Error bars at 13 days indicate the standard error of the mean.

TABLE I.—*Growth Delay Induced by Cyclophosphamide in Microscopic Tumours*

| Time of CY administration | Cyclophosphamide dose (mg/kg) 0 | 100 | 200 | 300 |
|---|---|---|---|---|
| 2 days after inoculation | 9, 9, 9, 9, 9, 9, 9, 9, 9, 10, 10, 10, 10, 10, 10, 12, 12, (9) | 15, 16, 16, 16, 16, 16, 16, 18, 18, 18, 19, 22, 23, 26, 29, 29, TC, (18) | 20, 20, 20, 20, 21, 21, 21, 22, 23, 23, 24, 26, 27, 27, 30, 35, TC, TC, TC, (22·5) | 19, 23, 24, 24, 24, 24, 24, 26, 27, 27, 31, 31, 33, 38, 38, TC, TC, (26) |
| 5 days after inoculation | as above | 11, 13, 13, 14, 14, 15, 15, 15, 15, 15, 15, 16, 16, 16, 17, 17, 17, 17, (15 | 17, 17, 17, 18, 18, 19, 19, 19, 19, 19, 20, 20, 21, 21, 21, 22, 22, 22, (19) | 20, 21, 22, 22, 22, 22, 23, 24, 24, 24, 24, 24, 24, 25, 26, 27, 27, 27, (24) |

Figures are times in days for individual tumours to reach 100 mm$^3$.
TC = animal alive and tumour-free at 100 days after treatment.
Figures in parentheses are the medians of the groups.

and the growth delay produced is almost exactly 3 days for each 100 mg/kg of CY. At no time after doses of 100 and 200 mg/kg does the tumour volume fall below that at the time of treatment, and for 300 mg/kg, the minimum volume is about 75% of the mean pretreatment value. For tumours treated at a size of 300 mm$^3$, the regrowth curves for 200 and 300 mg/kg were not parallel to the curve for untreated tumours. This was largely due to the fact that these high doses produced a necrotic region within the tumour extending towards the skin, with surface ulceration and subsequent contraction of the tumour mass. This pathology is typical of transplanted tumours growing intradermally where growth is lateral and to a depth, with a progressive deterioration of the vasculature to the central part of the tumour adjacent to the skin. The tumours do, however, then regrow rapidly. Similar curves were obtained in a repeat experiment. Between tumour volumes of 400 and 800 mm$^3$, where the departure from parallel regrowth is relatively small, the growth delay due to the CY is again very close to 3 days for each dose increment of 100 mg/kg.

*Microscopic tumours*

The growth delay induced by CY in microscopic tumours treated at 2 or 5 days after inoculation is shown in Table I. The data are pooled from 2 identical experiments. It may be seen that the growth delay for the microscopic tumours is greater than that for the larger tumours, *i.e.* about 5–6 days delay per 100 mg/kg of CY.

In vitro *plating of flank tumours*

The results of plating out cells from 50 mm$^3$ tumours at various times after administration of CY are shown in Fig. 2. Following a dose of 200 mg/kg (Fig. 2) the number of colonies per cell plated is reduced by more than $10^3$ when plating is carried out 2 h after drug administration. If, however, plating is delayed until 48 h after drug administration, the reduction in surviving fraction is only about ten-fold. Survival then gradually increases to reach normal levels 5–6 days after CY. The data for plating out following 100 or 300 mg/kg of CY again show a very large change in measured surviving fraction between 2 h and 48 h, being by a factor of around 15 at 100 mg/kg and around 100 at 300 mg/kg.

For tumours treated at 300 mm$^3$, the results are shown in Fig. 3. The initial (2-h) surviving fraction appears to be significantly higher at all 3 doses of CY than were seen for 50 mm$^3$. By 48 h, however, the values obtained are very similar to those seen for corresponding drug doses at the smaller tumour size.

FIG. 2.—Change in surviving fraction of cells from solid tumours with time between CY administration and preparation of cell suspension. Tumours treated at a volume of 50 mm$^3$. ○—100 mg/kg CY; ▲—200 mg/kg CY; ●—300 mg/kg CY. Horizontal dotted line shows lower limit of measurement in this assay system. Each point represents the mean value of surviving fraction for groups of 4 mice within the same experiment. Error bars show the standard error of the mean. The scale of the abscissa is broken.

FIG. 3.—Change in surviving fraction of cells from solid tumours with time between CY administration and preparation of cell suspension. Tumours treated at a volume of 300 mm$^3$. Otherwise as in Fig. 2.

The approximate factors between the 2-h and 48-h results are five-, ten- and twenty-fold at 100, 200 and 300 mg/kg respectively.

In one series of experiments at both tumour sizes, the procedure for making the cell suspension from solid tumours was particularly carefully controlled, in order to be as far as possible identical in all cases. The total number of viable tumour cells in the final suspension was determined for several tumours, both untreated and removed 2 or 48 h after CY (200 mg/kg). The mean cell yields were 4·6, 4·4 and 4·7 $\times$ $10^5$ cells/tumour respectively.

### In vitro *plating of lung tumours*

The data obtained for plating out cells obtained from tumour-bearing lungs at various times after treatment with CY are shown in Fig. 4. In these experiments, the CY was administered 9 days after tumour inoculation. For 100 mg/kg the mean value of a little below $10^{-3}$ obtained at 2 h rises to a mean value of about $5 \times 10^{-2}$ at 48 h, *i.e.* by a factor of 50–100. At 200 mg/kg the survival is below the lower limit of the technique (determined by fibroblast overgrowth if too many normal lung cells are plated into a dish), if measured at 2 h or 24 h after CY treatment, but has risen to a mean value of $5 \times 10^{-4}$ by 48 h.

### In vitro *plating: effect of medium*

Nine separate cell suspensions from tumours removed 2 h after treatment with

FIG. 4.—Change in surviving fraction of tumour cells growing in the lung with time between CY administration and preparation of cell suspension. Mice treated at 9 days after inoculation of tumour cells. ○—100 mg/kg CY; ▲—200 mg/kg CY. Horizontal dotted line shows lower limit of measurement in this assay system. Each point represents the mean value of surviving fraction for groups of 4 mice within the same experiment. Error bars show the standard error of the mean.

FIG. 5.—Dependence of time for tumours to reach a volume of 200 mm$^3$ on number of live cells inoculated on Day 0. Inocula of $10^5$ live cells or less were admixed with $10^6$ radiation-killed cells. The points represent the median values for groups of 8 mice. Two separate experiments are shown. The line is fitted by eye and corresponds to a tumour doubling time of 23 h.

CY (200 mg/kg) at a size of 50 mm$^3$ were plated into both depleted plateau medium and into fresh medium with a subsequent change of all plates to fresh medium after 48 h. The surviving fraction for fresh medium was 0·086 ± 0·023 (mean ± s.e.), whereas in plateau medium the surviving fraction was 0·28 ± 0·05, *i.e.* higher by a factor of 3·3. No difference was seen in the plating efficiency of cells from untreated tumours plated into the 2 media.

*Growth rate of microscopic tumours*

The times to reach a volume of 200 mm$^3$ for tumours growing from different numbers of live cells are shown in Fig. 5. The best line through the points indicates a doubling time for the tumour cell population of about 23 h.

*Cell loss*

The results of 2 separate experiments in which $^{125}$IUdR was used to study the effect of CY on cell loss are shown in Fig. 6.

A period of 48 h was allowed between administration of activity and first measurement, and the amount of label remaining in the acid soluble fraction should therefore have been only a few per cent.

It may be seen that the overall rate of loss of label is increased by the administration of CY, the activity remaining at

FIG. 6.—Loss of $^{125}$IUdR activity in solid tumours with time. Values are normalized to 100% at time 0 which was 48 h after administration of $^{125}$IUdR to tumours at a volume of 50 mm$^3$. Closed symbols—control mice; open symbols—mice receiving CY (200 mg/kg) at time 0. Different shape of symbols represent different experiments. Each represents the mean ± s.e. for a group of 5–8 mice.

FIG. 7.—Change in volume of solid tumours with time. ●—control tumours; ▲—mice given 2 doses of HU (each 1 mg/kg) separated by 3 h on Day 3 after tumour inoculation; △—dose separation = 7 h; ○—dose separation = 12 h. Each point represents the mean tumour volume for groups of 8 mice. Error bars shown at Day 15 represent the standard error of the mean.

6 days after treatment being about 25% with the drug and 45% without.

*Split-dose hydroxyurea: growth curves*

The effect upon tumour growth of a double dose of HU (each dose being 1 mg/g) given 3 days after tumour inoculation is shown in Fig. 7. With an interval of 3 h between doses, little growth delay is produced. An interval of 12 h produces a delay of about one day, but an interval of 7 h produces a delay of about 3 days. This result is in agreement with the findings of Hodgson *et al.* (1975) for the effect of split-dose HU on the survival of regenerating haemopoietic colony-forming cells in the spleens of BALB/c mice. The effect of a double dose of HU (each dose of 1 mg/g and separated by 7 h) upon control and regenerating tumours is shown in Fig. 8.

A double dose of HU given on the day upon which tumours reach 50 mm$^3$ has little effect upon tumour growth. If, however, the HU is given at 3 days after inoculation of the tumours, a delay of about 4 days compared with control tumours is produced. A dose of CY (200 mg/kg) given upon reaching 50 mm$^3$ produces a growth delay of about 5–6 days, which is in agreement with the results shown in Fig. 1. If the double dose of HU is given at 3 days after CY, an additional delay of around 2–3 days is produced. In Table II, the data from this experiment and 2 identical repeats

FIG. 8.—Change in volume of solid tumours with time. ●—control tumours normalized to have a volume of 50 $mm^3$ at Day 0; ○—as control, but receiving 2 doses of HU (each dose = 1 mg/g and separated by 7 h) on Day 0 (*i.e.* at a volume of 50 $mm^3$); ▲—as control, but receiving CY (200 mg/kg) on Day 0; △— as control but receiving CY (200 mg/kg) on Day 0 and 2 × HU on Day 3; ♦—a group inoculated with the tumour at the same time as the control group but receiving 2 × HU 3 days after tumour inoculation. Each point is the mean value of tumour volume for a group of 10 mice. Bars at 7 days show s.e. of mean.

are summarized. The growth delay has been quantified by measuring the difference in time to reach 200, 400 and 600 $mm^3$ and the mean value for the 3 experiments calculated. It may be seen that the value for 2 × HU at 3 days after CY lies approximately midway between the delay produced at 3 days after inoculation and at 50 $mm^3$ for untreated tumours.

### DISCUSSION

It is very clear from the data presented here for the *in vitro* plating assay after treatment of the EMT6 tumour with cyclophosphamide, that a wide range of estimates of surviving fractions may be obtained, depending upon the time selected for assay. As an example we may consider the response of 50-$mm^3$ tumours to 200 mg/kg, where the mean surviving fractions are $\sim 5 \times 10^{-4}$ at 2 h, $2 \times 10^{-3}$ at 24 h and $4 \times 10^{-2}$ at 48 h. A factor of about 100 is therefore seen between 2 and 48 h. There is no significant difference in cell yield from tumours at these times, and furthermore the data for $^{125}$IUdR loss indicates only a small difference in removal of label from untreated and CY-treated tumours. We can therefore regard the selective loss of CY-killed cells from the tumour over a period of 48 h as a minimal factor. Selective proliferation of non-killed cells is an alternative factor which could influence the result

TABLE II.—*Tumour Growth Delay Produced by Split-Dose Hydroxyurea*

| Growth delay estimated at a size of ($mm^3$) | Tumours treated at 50 $mm^3$ | | | Tumours treated at 3 days after inoculation | | | Tumours treated at 3 days after CY (200 mg/kg) given at 50 $mm^3$ | | |
|---|---|---|---|---|---|---|---|---|---|
| | 200 | 400 | 600 | 200 | 400 | 600 | 200 | 400 | 600 |
| Experiment A | 0·0 | −1·5 | −1·1 | 4·5 | 3·0 | 3·2 | 1·3 | 1·0 | 2·5 |
| Experiment B | 1·2 | 1·2 | 2·0 | 3·4 | 4·8 | 5·3 | 3·2 | 2·4 | 1·9 |
| Experiment C | 1·3 | 1·2 | 0·2 | 4·6 | 4·4 | 5·0 | 1·2 | 3·0 | 2·0 |
| Mean delay (3 experiments estimated at 3 different sizes) | | 0·5 | | | 4·2 | | | 2·1 | |

All growth delays given in days.
Hydroxyurea given in 2 doses each of 1 mg/g with an interval of 7 h between doses.

and which would not necessarily be detectable by significant changes in the cell yield, as only a few per cent of the tumour cell population need be involved. We have, however, to explain an increase by a factor of 100 over 48 h. The doubling time of untreated 50-mm$^3$ tumours is 40–50 h. This is, however, much longer than the cell cycle time which, from the available data for other EMT6 sublines (Rockwell *et al.*, 1972; Watson, 1976) probably lies in the range of 16 to 19 h, with a growth fraction of $\sim 50\%$ and significant cell loss. If it is assumed that the surviving clonogenic cells are a representative fraction of the total number of clonogenic cells, then it is difficult to see why the doubling time of the survivors should be any shorter than that of the untreated tumour at a given size. This might occur if there were a sudden large-scale cell loss and rearrangement of tumour structure, but this does not appear to happen. If, on the other hand, the surviving cells are able to respond to the new reduced size of their own population, they may do so by doubling in a manner akin to that of tumours growing from a small inoculum in the presence of radiation- (or drug-) killed cells. In this situation, as shown in Fig. 5, the doubling time is about 23 h. If the experiment using 2 doses of HU and observing the growth-curve delay is valid, it appears that the doubling time of surviving cells (measured at 3 days after CY) lies somewhere between these 2 possibilities. If, however, for the sake of argument we regard the lower estimate of 23 h as the shortest realistic doubling time and further assume that

(a) doubling of surviving cells begins essentially immediately after CY treatment, *i.e.* no growth delay induced by the drug, and

(b) accelerated growth of survivors continues right up until the time that the pretreatment tumour volume is reached; the maximum number of doublings occurring during the period of 6 days growth delay after 200 mg/kg is about 6, which is equivalent to a surviving fraction of around 1·5%. Furthermore, the increase over the first 48 h can only be by a factor of 4. Both these figures are incompatible with the data for *in vitro* plating. It is possible that the regrowth of tumours after CY treatment is accelerated by immune depression of the host. We do not believe this to be true, however, as there is no difference in the rate of growth of the EMT6 tumour in animals treated with CY before inoculation and in untreated controls.

We are therefore left with the possibility that repair of potentially lethal damage (PLD) (as suggested by Hahn *et al.*, 1973) is in part responsible for the change in observed surviving fraction between 2 and 48 h. In their original study of the EMT6 tumour, Hahn *et al.* (1973) found a difference by a factor of 4–5 in the measured surviving fraction at 2 and 24 h after 200–300 mg/kg of CY. Using the B16 melanoma, however, Hill and Stanley (1975) found no difference in surviving fraction measured 2 or 22 h after the drug. In the Lewis lung tumour, Steel and Adams (1975) used times of 3 h and 16 h after CY, and found no difference between these. We must therefore conclude either that EMT6 is the only one of these three different tumours to carry out PLD repair after CY, or else that for B16 and Lewis lung the PLD repair occurs between 24 and 48 h after drug treatment. There is certainly a strong indication from the data presented in this paper that PLD repair in the EMT6 tumour continues after 24 h.

The effect of this early increase in measured surviving fraction following CY is very important when considering the relative effect of the drug upon different tumour sizes. It may be seen that the surviving fraction measured at 2 h is significantly lower in 50-mm$^3$ than in 300-mm$^3$ tumours, for all drug doses. By 48 h, however, the differential is largely lost, due apparently to a greater repair of PLD in the smaller tumours. This finding is in agreement with the observation that the growth delay induced by the same dose of CY is similar at 2 tumour sizes. For tumour cells growing in the lungs, the response measured at either 2 h or 48 h appears to be somewhat greater than in the solid tumour. In our previous study, in which we looked at lower doses of CY (up to 140 $\mu$g/ml) we did not see much difference between the response of small tumours and lung nodules measured 2 h after CY. The spread of results in this type of assay is, however, sufficiently great to make the 2 results compatible. The delay in tumour growth produced by CY given to animals bearing microscopic tumours is, however, greater than that seen in the macroscopic tumours, adding weight to the argument that a degree of tumour-size dependence occurs with CY.

Whichever is the true value for the surviving fraction of cells in solid tumours after CY treatment, it is clear that the change in tumour size is a very poor guide to this. Even after 300 mg/kg at 50 mm$^3$ the maximum reduction in tumour volume is 25%, and at lower doses no reduction at all occurs. This is probably due to the short time course of regeneration of surviving cells compared with the rate of cell loss following drug treatment.

The points which we have made regarding the nature of " PLD " following treatment of the EMT6 tumour with bleomycin (Twentyman and Bleehen, 1975) apply largely to cyclophosphamide also. The expression " repair of PLD " refers only to the observed phenomenon of increased surviving fraction with delayed subculture rather than carrying any implication regarding mechanisms. It is clear that the effect following CY occurs over a longer period than that following bleomycin or X-irradiation (Little *et al.*, 1973; Hahn *et al.*, 1974) and also that the effect occurs to at least as great an extent in small tumours as in large ones.

With " PLD repair " extending over a period of at least 48 h, by which time the proliferation of surviving cells is probably well under way, there is no time at which the absolute value of " surviving fraction " can correctly be measured. Unless some way of inhibiting either PLD repair or proliferation of survivors, but not both, is found, the time at which " surviving fraction " determinations are made is largely arbitrary. It seems to us, at this stage, that " growth delay " may be the most relevant basis for comparing the effectiveness of various treatment modalities upon the EMT6 tumour. We need, however, to collect much more data following treatment with different agents, before firm conclusions can be reached.

I wish to thank Professor N. M. Bleehen for his continued support and for discussion of the data, and Mrs Jane Donaldson for her expert technical assistance.

### REFERENCES

BROWN, J. M. (1974) Radiosensitization of Pulmonary Metastases with Intravenous Infusion of Pyrimidine Analogs. Paper read at 5th International Congress of Radiation Research, Seattle, U.S.A.

HAHN, G. M., RAY, G. R., GORDON, L. F. & KALLMAN, R. F. (1973) Response of Solid Tumor Cells to Chemotherapeutic Agents *in vivo*. Cell Survival after 2 and 24 hour Exposure. *J. natn. Cancer Inst.*, **50**, 529.

HAHN, G. M., ROCKWELL, S., KALLMAN, R. F., GORDON, L. F. & FRINDEL, E. (1974) Repair of Potentially Lethal Damage *in vivo* in Solid Tumor Cells After X-Irradiation. *Cancer Res.*, **34**, 351.

HILL, R. P. & STANLEY, J. A. (1975) The Response of Hypoxic B16 Melanoma Cells to *in vivo* Treatment with Chemotherapeutic Agents. *Cancer Res.*, **35**, 1147.

HODGSON, G. S., BRADLEY, T. R., MARTIN R. F., SUMNER, M. & FRY, P. (1975) Recovery of Proliferating Haemopoietic Progenitor Cells after Killing by Hydroxyurea. *Cell Tissue Kinet.*, **8**, 51.

LITTLE, J. B., HAHN, G. M., FRINDEL, E. & TUBIANA, M. (1973) Repair of Potentially Lethal Radiation Damage *in vitro* and *in vivo*. *Radiology*, **106**, 689.

ROCKWELL, S. C., KALLMAN, R. F. & FAJARDO, L. F. (1972) Characteristics of a Serially Transplanted Mouse Mammary Tumor and its Tissue-culture-adapted Derivative. *J. natn. Cancer Inst.*, **49**, 735.

STEEL, G. G. & ADAMS, K. (1975) Stem-cell Survival and Tumour Control in the Lewis Lung Carcinoma. *Cancer Res.*, **35**, 1530.

TWENTYMAN, P. R. & BLEEHEN, N. M. (1974) The Sensitivity to Bleomycin of a Solid Mouse Tumour at Different Stages of Growth. *Br. J. Cancer*, **30**, 469.

TWENTYMAN, P. R. & BLEECHEN, N. M. (1975) Studies of "Potentially Lethal Damage" in EMT6 Mouse Tumour Cells Treated with Bleomycin either *in vitro* or *in vivo*. *Br. J. Cancer*, **32**, 491.

TWENTYMAN, P. R. & BLEEHEN, N. M. (1976) The Sensitivity to Cytotoxic Agents of the EMT6 Tumour *in vivo*. Comparative Response of Lung Nodules in Rapid Exponential Growth and of the Solid Flank Tumour. *Br. J. Cancer*, **33**, 320.

WATSON, J. V. (1976) The Cell Proliferation Kinetics of the EMT6/M/AC Mouse Tumour at Four Volumes During Unperturbed Growth. *Cell Tissue Kinet.*, **9**, 147.